# Diagnosis and management of bovine babesiosis outbreaks in cattle in Punjab state

**DOI:** 10.14202/vetworld.2016.1370-1374

**Published:** 2016-12-06

**Authors:** Mandeep Singh Bal, Vishal Mahajan, Gursimran Filia, Paramjit Kaur, Amarjit Singh

**Affiliations:** 1 Animal Disease Research Centre, Guru Angad Dev Veterinary & Animal Sciences University, Ludhiana, Punjab, India; 2 Department of Veterinary Parasitology, Guru Angad Dev Veterinary & Animal Sciences University, Ludhiana, Punjab, India

**Keywords:** *Babesia bigemina*, cattle, outbreaks, parasitological diagnosis, polymerase chain reaction

## Abstract

**Aim:**

The aim of the present study was to diagnose severe outbreaks of bovine babesiosis in Punjab state, in the year 2015 and to suggest control and preventive measures to animal owners.

**Materials and Methods:**

Mortality of animals was recorded in two cattle herd comprising a total of 465 cattle in Sangrur (n=125) and Faridkot (n=340) districts. There was a history of purchase of animals at one farm. 23 blood samples were collected from diseased (n=15) and healthy animals (n=8) for hematological analysis, parasitological, and polymerase chain reaction (PCR)-based diagnosis. Ticks were also collected from animals for identification.

**Results:**

Out of 465 cattle at risk, 28 were critically ill and 14 died of disease with morbidity, mortality, and case fatality rate of 6.02%, 3.01%, and 50.00%, respectively. Clinical signs and necropsy findings were suggestive of babesiosis. Ticks collected from both the outbreaks were identified as *Rhipicephalus (Boophilus) microplus*. Thin blood smears from infected animals (especially with clinical sign of hemoglobinuria) were found positive for *Babesia bigemina* organisms; however, molecular diagnosis (PCR) further confirmed the disease. Animals were successfully treated with diminazene aceturate, hematinics, and antipyretics.

**Conclusions:**

Two fatal outbreaks of babesiosis in cattle were diagnosed with application of conventional parasitological, hematological, and molecular diagnostic techniques. PCR was found to be far more sensitive in detecting the disease, especially in latent infections. Animal owners were advised to follow quarantine measures before mixing new animals in the herd and strategic acaricidal treatments for effective tick control.

## Introduction

Bovine babesiosis caused by intraerythrocytic hemoprotozoa *Babesia bigemina* is a tick-borne disease affecting the bovines in tropical and subtropical parts of Africa, Australia, America, and Asia including India. Walker and Edward [1] first time reported babesiosis in India. Annual economic losses to livestock due to babesiosis in India are estimated to be about 57.2 million US dollars [2].

Punjab (situated in the North-western part of India), the third highest milk producing state of the country, has a total of 6.7 million cattle and buffalo population as per the 18^th^ livestock census (http://www.husbandrypunjab.org/pages/livestock.html). The climate conditions of Punjab state are conducive for tick vector survival. Clinically, disease has been characterized by anemia, fever, hemoglobinuria, and in many cases death [3]. The crossbred cattle exhibited higher rate of susceptibility than zebu and buffaloes, which mainly act as carrier [4]. In buffaloes, the clinical symptoms reported are hemoglobinuria, anorexia, suspended rumination, reduced milk yield, and depression [5-7]. The calves up to 9-12 months of age are generally resistant due to inverse age resistance, but the clinical symptoms of babesiosis in neonatal calves were inability to suckling, high fever, coffee color urine, jaundice, and deep shallow respiration [8,9].

The classical microscopic examination of *Babesia* piroplasms in Giemsa stained thin blood smear is a gold standard test that is relatively cheap and quick method; however, in chronic infection, it has low sensitivity and usually fails to detect carrier animals [10].

In Indian scenario, there are sporadic reports on diagnosis of bovine babesiosis by conventional parasitological methods [11-13]. Serological tests are used for large-scale epidemiological studies. However, they fail to detect early infection. Alternatively, polymerase chain reaction (PCR) has been widely used for the detection of *Babesia* parasites owing to its high specificity and sensitivity [14,15]. The present reports depict the confirmatory diagnosis of two severe outbreaks of bovine babesiosis based on conventional microscopy and PCR targeting the small subunit ribosomal RNA (SSU rRNA) sequence of *B. bigemina* and its management.

## Materials and Methods

### Ethical approval

The manuscript is related to the investigation of outbreaks based on the urgent request by the livestock farmers/animal husbandry department official to rule out the cause. Hence it is not the experimental study, as per institution guidelines ethical approval is not required.

### Animals and sampling

This department received requests from local veterinarians/farmers regarding mortality of animals (cattle) with symptoms of high fever and red/coffee-colored urine in Sangrur and Faridkot districts of Punjab. The two cattle herd comprising total 465 cattle in Sangrur (n=125) and Faridkot (n=340) where mortality of animals was recorded. Animals of both of the farms were stall-fed. Deworming and routine vaccination practices were followed at farms. Tick infestation was also observed in animals. Twenty-three blood samples were collected from diseased (n=15) and healthy animals (n=8) for hematological analysis, parasitological, and molecular diagnosis. Approximately 5 ml of blood sample from jugular vein was collected in ethylenediaminetetraacetic acid coated and plain vacutainers from each animal. Thin blood smears were prepared and stained with Leishman stain. There was a history of purchase of animals at one farm in district Sangrur. Outbreaks were reported in the months of June (Sangrur) and July (Faridkot).

### Sample analysis

Hematological parameters, namely, total leukocytes count (10^3^ cells/µL), hemoglobin (Hb, g/dL), packed cell volume (%), differential leukocyte count (%), and platelets (Plt, 10^3^ cells/µL) were evaluated in parasitologically positive animals (Group I, n=7), parasitologically negative (Group II, n=8), and healthy control (Group III, n=8) animals. Ticks were collected from animals of both the farms, mounted in Canada balsam after clearing as per standard procedure and identified microscopically according to keys given by Miranpuri [16].

Genomic DNA was extracted from all the collected blood samples using HiPurA™ blood Genomic DNA MiniPrep Purification Spin Kit as per the given protocol (HiMedia Laboratories, India). PCR was carried out on the DNA targeting the SSU rRNA of *B. bigemina* [17]. PCR was carried out in reaction mixture (25 µl) contained 12.5 µl of KAPA 2G™ Fast HotStart Ready Mix (2× containing KAPA 2G Fast HotStart DNA polymerase, KAPA 2G Fast HotStart PCR buffer, 0.2 mMdNTP each, 1.5 mM MgCl_2_), 1.5 µl of 10 pmol primers each, 4.5 µl of NFW, and 5 µl of DNA template. Amplification was carried out with cycling conditions: initial denaturation at 95°C for 5 min, 30 cycles of denaturation at 95°C for 30 s, annealing at 57°C for 1 min, extension at 72°C for 1.5 min, and final extension at 72°C for 10 min in a thermocycler (Eppendrof, Germany). The amplified PCR products (689 bp) were visualized using 1.5% agarose gel electrophoresis (Syngene, UK).

### Treatment

Treatment of infected animals was carried out and control measures were suggested to animal owners. Officials of animal husbandry department/local veterinarians were also informed about the outbreaks for implementation of control measures to prevent the spread of disease among other animals in the surrounding areas.

### Statistical analysis

Data were analyzed by analysis of variance using SPSS software.

## Results

**Figure-1:**
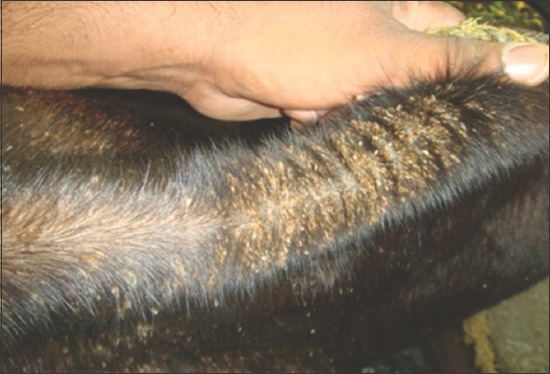
Heavy tick infestation.

Out of total 465 cattle at risk (125 Sangrur and 340 Faridkot), 28 (12 Sangrur and 16 Faridkot) were critically ill and 14 (6 Sangrur and 8 Faridkot) died of disease with morbidity, mortality, and case fatality rates of 6.02%, 3.01%, and 50.00%, respectively. Major clinical symptoms observed in affected animals were pale mucous membranes, jaundice, increased respiratory rate, hemoglobinuria, and fever. Moderate to heavy tick infestation was observed in animals (Figure-1). Ticks collected from both the outbreaks were identified as *Rhipicephalus (Boophilus) microplus*.

**Figure-2:**
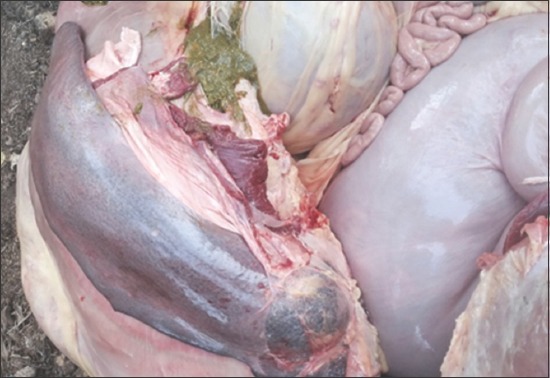
Splenomegaly and jaundice.

**Figure-3:**
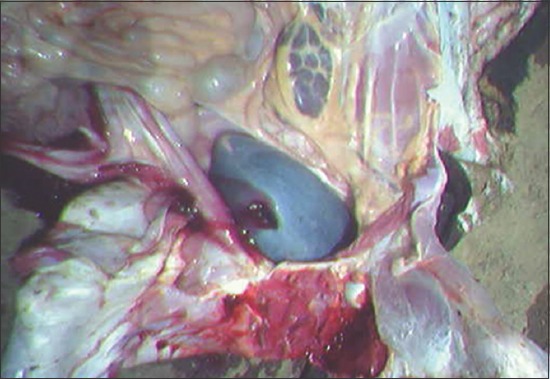
Red/coffee-colored urine in urinary bladder of infected cattle died of disease.

Postmortem of two animals (one at each farm) was also conducted on the spot. Necropsy findings revealed splenomegaly, epicardial hemorrhages, thick granular bile, and urinary bladder filled with coffee-colored urine (Figures-2 and 3).

**Figure-4:**
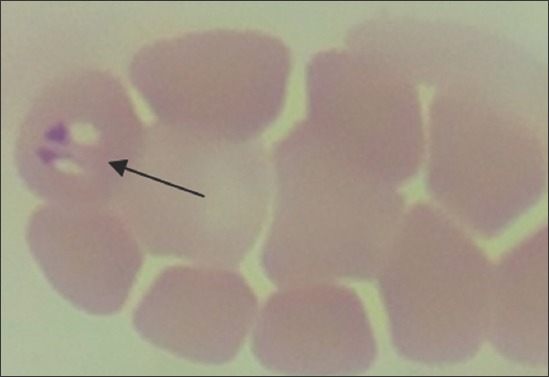
*B. bigemina* piroplasm in cattle blood: Leishman stained thin blood smear (100×).

**Figure-5:**
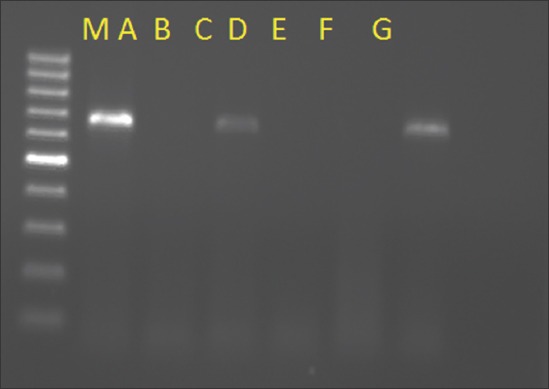
Agarose gel (1.5%) electrophoresis showing 689 bp fragment of amplified *B. bigemina* in blood samples. Lane M: Molecular marker 100 bp, Lane A: Positive control, Lane B: Host leukocytes DNA, Lane C-F: Tested samples, Lane G: Negative control.

Stained blood smears were examined for hemoparasites. Out of 15 diseased animals, 7 samples were found positive for piroplasms of *B. bigemina* (Figure-4). Out of these 7 animals, 6 were having history of passing red-colored urine. PCR was employed on DNA extracted from all the 23 collected samples (15 diseased and 8 healthy animals). Out of 23 samples, 13 were found positive (including 7 microscopically positive) for *B. bigemina* DNA as evident from agarose gel (1.5%) electrophoresis showing 689 bp fragment of amplified DNA/PCR product (Figure-5). It indicates a higher sensitivity of PCR over traditional blood smear examination, especially for detecting latent infections. None of the sample from healthy animals was amplified by PCR. Thin blood smears from infected animals with clinical sign of hemoglobinuria were found positive for *B. bigemina* organisms; however, positive results by molecular diagnosis (PCR) further confirmed the disease.

The hematological parameters showed a significant (p<0.05) decrease in Hb (3.5±0.197 g/dl) in Group I animals (parasitologically positive) as compared to Group II (7.79±0.252 g/dl) and Group III (9.89±0.234 g/dl) (Table-1). The leukocytosis was observed in animals of Group I as compare to Group III animals being non significant.

**Table-1:** Hematological parameters in parasitologicaly positive (Group I), parasitologicaly negative (Group II) and healthy control (Group III) animals.

Group	Hb (g/dl)	TLC (per µl)	DLC (%)			

			N (%)	L (%)	M (%)	E (%)
I (n=7)	3.5*±0.197	12.44±0.464	39.38±0.891	58.39±0.234	1.28±0.187	0.70±0.138
II (n=8)	7.79±0.252	9.85±0.243	37.90±0.128	59.55±0.464	1.38±0.494	0.88±0.123
III (n=8)	9.89±0.234	10.77±0.470	40.34±0.236	57.14±0.125	1.18±0.324	0.78±0.112

* Statistically significant (p<0.05) as compare to Groups II and III. Hb=Hemoglobin, TLC=Total leucocytes count, DLC=Differential leucocyte count

Affected animals were successfully treated with diminazene aceturate, hematinics, and antipyretics. However, despite treatment, one animal died of disease due to advanced stage of disease.

## Discussion

The predominant symptoms of babesiosis; pale mucus membranes, jaundice, increased respiratory rate, hemoglobinuria, and fever were observed in parasitologically positive animals which are in agreement of published reports [11,18,19].

The marked anemia and hemoglobinuria in cattle leads to the severe hemolytic process associated with the presence of *Babesia* piroplasms inside the erythrocytes and destruction of large number of these erythrocytes by the parasite resulting in hemoglobinemia and consequently hemoglobinuria [20].

In Punjab, the *R. (B.) microplus* is the predominant tick responsible for the disease [15] and identification of collected ticks from diseased animals supports the same. The vector widely known for the transmission of bovine babesiosis is *R*. (*B*.) *microplus;* however, transmission by *Hyalomma anatolicum anatolicum* was also reported [21].

Conventional diagnostic method, namely, stained blood smear examination revealed only 7 animals positive for babesiosis out of 15 diseased animals. The direct method of identifying the parasite in Giemsa-stained blood smears is the gold standard test for diagnosis of babesiosis; however, this technique shows a low sensitivity in subclinical and chronic phase of the infection [22]. In addition, six cattle found positive by PCR were not having the predominant signs of babesiosis except weakness indicated the subclinical infections. Recently, PCR was employed to rule out the latent infection of the bovine babesiosis from Punjab [15]. Fahrimal *et al*. [23] and Zulfiqar *et al*. [24] reported greater sensitivity and specificity of PCR assays over the existing tests for diagnosis of bovine babesiosis.

Outbreaks were recorded in the month of June and July that are the favorable months for multiplication of the tick vector (*R*. [*B*.] *microplus)* identified in the present study. (*R. (B.) microplus*). Singh *et al*. [25] reported *R. (Boophilus) microplus* as predominant tick species in cattle and buffaloes of Punjab. There was a history of purchase of new animals in Sangrur district. Further, proper quarantine measures were not followed at the farm. It could be the possibility that newly purchased animals (in the carrier stage/incubation phase of disease) may be responsible for outbreak at the farm.

Livestock owners were advised to follow quarantine measures before intruding new animal into the herd. They were advised for tactical acaricidal treatments for effective tick control. Moreover, they were also advised for rotation of drugs and to administer proper dosage to prevent acaricidal resistance. Officials of Animal Husbandry Department were also informed about the outbreaks for implementation of control measures to prevent the spread of disease among other animals in the surrounding areas.

## Conclusion

Two fatal outbreaks of babesiosis in cattle were diagnosed with application of conventional parasitological, hematological, and molecular diagnostic techniques. PCR was found to be far more sensitive in detecting the disease, especially in latent infections. Diseased animals were successfully treated and animal owners were advised to follow preventive measures for tick control.

## Authors’ Contributions

MSB, VM visited the farms for sample collection, carried out parasitological and hematological analysis in the laboratory. GF, PK and MSB carried out molecular analysis of samples. AS helped in planning, statistical analysis and supervised the research work. All authors drafted, revised and approved the final manuscript.
